# State-dependent diffusion coefficients and free energies for nucleation processes from Bayesian trajectory analysis

**DOI:** 10.1080/00268976.2018.1471534

**Published:** 2018-05-13

**Authors:** Max Innerbichler, Georg Menzl, Christoph Dellago

**Affiliations:** a Faculty of Physics and Center for Computational Materials Science, University of Vienna, Vienna, Austria; b Faculty of Physics, University of Vienna, Vienna, Austria

**Keywords:** Bayesian inference, diffusion, nucleation, classical nucleation theory, cavitation

## Abstract

The rate of nucleation processes such as the freezing of a supercooled liquid or the condensation of supersaturated vapour is mainly determined by the height of the nucleation barrier and the diffusion coefficient for the motion across it. Here, we use a Bayesian inference algorithm for Markovian dynamics to extract simultaneously the free energy profile and the diffusion coefficient in the nucleation barrier region from short molecular dynamics trajectories. The specific example we study is the nucleation of vapour bubbles in liquid water under strongly negative pressures, for which we use the volume of the largest bubble as a reaction coordinate. Particular attention is paid to the effects of discretisation, the implementation of appropriate boundary conditions and the optimal selection of parameters. We find that the diffusivity is a linear function of the bubble volume over wide ranges of volumes and pressures, and is mainly determined by the viscosity of the liquid, as expected from the Rayleigh–Plesset theory for macroscopic bubble dynamics. The method is generally applicable to nucleation processes and yields important quantities for the estimation of nucleation rates in classical nucleation theory.

## GRAPHICAL ABSTRACT

**Figure:**
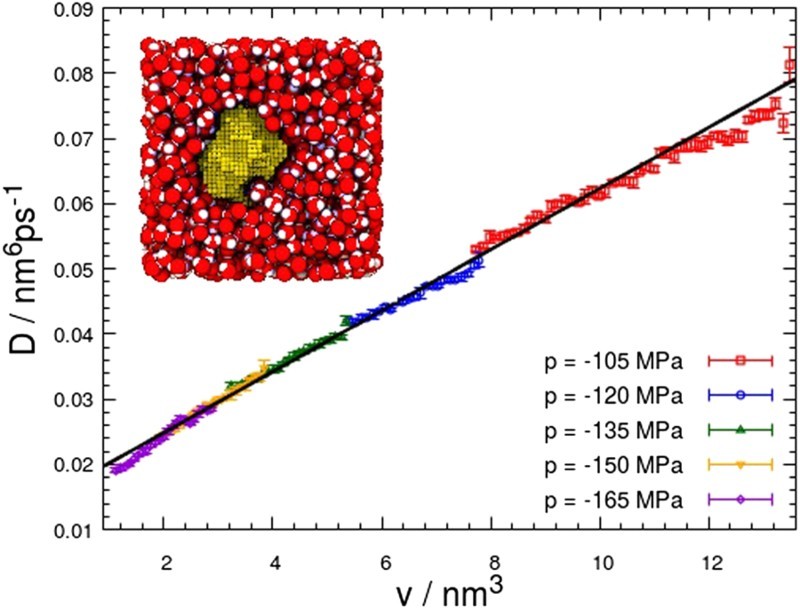

## 1. Introduction

The mechanism and kinetics of first-order phase transitions can be conceptually understood in the framework of classical nucleation theory (CNT). In this model, the phase transition occurs via the formation of a small nucleus of the new, thermodynamically favoured phase within the old phase. Initially, growth of the nucleus is impeded by a free energy barrier arising from the cost of creating an interface between the two phases. For larger nuclei, however, this free energetic cost is outweighed by the favourable contribution of the new phase. As a consequence, the thermodynamically stable phase evolves to macroscopic scales only if the nucleus grows to the so-called critical size due to a rare thermal fluctuation.

The statistical character of the nucleation process is captured by Kramers' theory of barrier crossing [1,2], in which one imagines that the system evolves stochastically along a reaction coordinate *q* under the influence of a free energy G(q) and a state-dependent diffusivity D(q) (frequently replaced, however, by assuming a uniform diffusion constant *D*). While the kinetics of nucleation is in large part governed by the free energy landscape G(q) and much work has been done to determine nucleation free energies using computer simulations [3–5], also the diffusivity D(q) plays a fundamental role in predicting how the nucleation process unfolds [3,6].

In this work, we simultaneously calculate the nucleation free energy and state-dependent diffusion coefficient near the nucleation barrier using a Bayesian analysis approach devised by Hummer [7]. In this method, which assumes Markovian dynamics, a rate matrix is introduced that describes the kinetics of transitions between discretised bins of a given reaction coordinate. The rate matrix, from which both the free energy profile G(q) and the diffusivity D(q) can be determined, is adapted to reproduce the dynamics observed in dynamical trajectories obtained from simulations as closely as possible. In applying this procedure, particular attention needs to be paid to the effects of discretisation on systematic and statistical errors arising from a limited set of input data.

**Figure 1.**
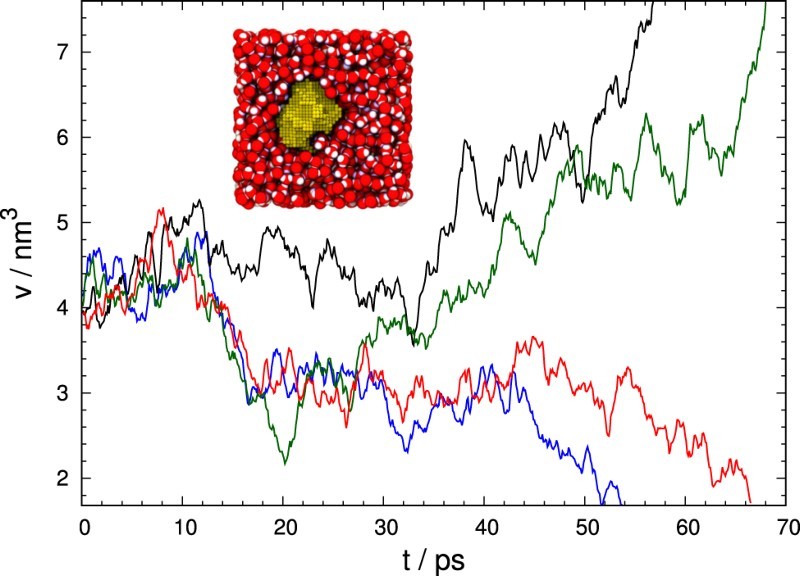
Examples for the time evolution of the largest bubble volume, *v*, in cavitating water at a pressure of p=−135MPa. The trajectories, obtained with molecular dynamics (MD) simulations [8], start from equilibrium configurations near the top of the nucleation barrier. After leaving the proximity of the maximum, *v* tends to shrink or grow swiftly, determining whether the system subsequently reaches the metastable liquid or relaxes to the vapour phase. A snapshot taken from an MD simulation is shown in the inset.

We apply this method to analyse the free energy and dynamics of cavitation in liquid water under tension, i.e. at negative pressures [8–14]. While liquid water under tension is metastable, it can sustain negative pressures exceeding −120MPa for long periods due to the strong cohesion between water molecules [15]. Eventually, however, vapour bubbles will nucleate and the system relaxes to the vapour phase. For cavitation, the size of the largest bubble has been shown to be a good reaction coordinate capable of capturing the essential transition mechanism [8,16,17]. The volume of the largest bubble is a collective variable and the influence of many underlying degrees of freedom gives rise to diffusive dynamics (as illustrated in Figure 1). By applying Hummer's Bayesian analysis approach to dynamical barrier crossing trajectories obtained earlier from molecular simulations [8], we compute the diffusivity over a wide range of bubble sizes for various pressures. We find that the diffusivity depends linearly on bubble volume and is mainly determined by the viscosity of the liquid as predicted by the Rayleigh–Plesset equation, which describes the dynamics of a macroscopic gas-filled bubble in an incompressible fluid [18]. In addition to the diffusion coefficient, our analysis also yields the free energy profile near the top of the barrier. Its curvature is related to the so-called Zeldovich factor, which encodes the dynamics of nucleus growth in CNT and is needed for the calculation of nucleation rates, for instance in the seeding method [6].

The remainder of the article is organised as follows. In Section 2 the Bayesian approach of Hummer is briefly reviewed. The method is then tested in Section 3 for synthetic data generated with a simple one-dimensional model of nucleation. Here, we focus especially on discretisation effects and the optimal selection of parameters. In Section 4, we extract free energy and diffusion profiles from simulation data, followed by our conclusions in Section 5.

## 2. Bayesian inference of rate matrices

To extract the diffusivity and free energy landscape from MD trajectories, we employ an algorithm developed by Hummer based on Bayesian inference [7]. As a starting point consider a one-dimensional Fokker–Planck equation [19], which describes the stochastic dynamics of a system subjected to dragging forces and diffusion in terms of a time-dependent probability density p(q,t),

(1) $$\partial_{t}p(q,t)=\partial_{q}\left[D(q)\;e^{-\beta G(q)}\partial_{q}\left[e^{\beta G(q)}p(q,t)\right]\right].$$

Here, D(q) and G(q) denote the diffusion coefficient and the free energy, respectively, both of which are a function of the reaction coordinate *q*. One then discretises this equation by imposing a grid consisting of *n* bins of equal width, Δq, on the reaction coordinate. The centre of bin *j* is denoted as $q_{j}$, and we write $f_{j}=f(q_{j})$ for arbitrary functions f(q). A possible spatial discretisation of the Fokker–Planck equation (1) reads [20]

(2) $$\begin{array}{rl} \partial_{t}p_{j}(t) & =R_{j,j-1}p_{j-1}(t)+R_{j,j+1}p_{j+1}(t)\\ & \;-(R_{j-1,j}+R_{j+1,j})p_{j}(t)+O(\Delta q^{2}), \end{array}$$

where the rate coefficients $R_{ij}$ are related to the diffusion constant and the probability density by

(3) $$R_{j,j\pm 1}=\frac{D_{j}+D_{j\pm 1}}{2\Delta q^{2}}{\left(\frac{p_{j}}{p_{j\pm 1}}\right)}^{1/2}.$$

Note that while the reaction coordinate *q* is now discretised, time *t* is still continuous. Equation (2) has the form of a master equation for a discrete Markovian system

(4) $${\dot{p}}_{i}(t)=\sum_{j}R_{ij}p_{j}(t),$$

which governs the time evolution of the probabilities $p_{i}(t)$ to find the system in bin *i*.

The master equation (4) can be formally solved in terms of a matrix exponential

(5) $$p_{i}(t)=\sum_{j}(e^{tR}{)}_{ij}p_{j}(0).$$

In order to conserve probability, $e^{tR}$ needs to satisfy all properties of a stochastic matrix. If the stochastic matrix is irreducible, a unique equilibrium distribution $p_{j}^{\mathrm{eq}}$ exists, corresponding to eigenvalue one, and related to the free energy via $G_{j}=-\beta \ln p_{j}^{\mathrm{eq}}$. Starting from these expressions, we proceed to outline the general idea of the Bayesian inference algorithm employed here to determine the rate coefficients $R_{ij}$ that best fit a set of empirical data.

This goal can be achieved by applying Bayes' theorem, which relates conditional and marginal probabilities,

(6) P(A|B)P(B)=P(B|A)P(A).

Here, *A* and *B* each indicate an arbitrary event. In the following, we will identify *A* with a set of parameters, i.e. the elements of the rate matrix $R_{ij}$, and *B* with empirical data extracted from simulated trajectories. More specifically, by slicing a trajectory in steps of a selected lag time *τ* we count the number of transitions $N_{ij}(\tau )$ from bin *j* to bin *i* occurring during a particular simulation. The lag time *τ* should be chosen large enough so that the dynamics expressed in terms of such transitions is Markovian. Given a sufficiently large amount of data, either from a single long equilibrium trajectory or numerous short ones, one obtains a statistically significant matrix of transition events, $\left\{N_{ij}\right\}$. The columns of this matrix represent the transition histogram of the respective bin.

Comparing the matrix of transition events, $\left\{N_{ij}\right\}$, with the formal solution, Equation (5), it is evident that individual empirical transition probabilities $N_{ij}/\sum_{i}N_{ij}$ should approximate the elements of the matrix exponential, i.e. $p(j\to i,\tau )=(e^{\tau R}{)}_{ij}$. Provided that transition events are statistically independent, the likelihood *L* to observe a certain set of data given some rate coefficients can be expressed as a product

(7) $$L=P(N_{ij}\;|\;R_{ij},\tau )=\prod_{i,j}(e^{\tau R}{)}_{ij}^{N_{ij}}.$$

Bayes' theorem then implies $P(R_{ij}\;|\;N_{ij},\tau )\propto P(N_{ij}\;|\;R_{ij},\tau )P(R_{ij})$.

Typically, the marginal distribution $P(R_{ij})$ of the parameters is not known a priori, but may be utilised as a means to introduce bias to a simulation. For instance, one can use it to impose continuity, or to bias parameters to stay close to some reference estimates. In the simplest implementation of the approach, however, one may assume a uniform distribution [7]. By maximising the scalar likelihood function *L* in the space of rate matrices, one obtains estimates for $D(q_{j}+\Delta q/2)$ and $G(q_{j})$ via Equation (3). This maximisation can be carried out by steepest descent methods or similar algorithms, but also by Markov chain Monte Carlo (MCMC) sampling [21].

In a one-dimensional problem such as the one considered here, the number of independent parameters is considerably reduced since *R* fulfils the following conditions:

(8) $$R_{ij}=\left\{\begin{array}{ll} R_{ij} & \mathrm{if}\;i>j,\\ -\sum_{i(\neq j)}R_{ij} & \mathrm{if}\;i=j,\\ R_{ji}\frac{p_{i}}{p_{j}} & \mathrm{if}\;i<j. \end{array}\right.$$

The second relation in Equation (8) emerges from total probability conservation, making the exponential a stochastic matrix. The third one is a direct result of the transition rates obeying detailed balance (see Equation (3)). Furthermore, Equation (2) implies that *R* is tridiagonal for reflective or absorbing boundary conditions, as the system evolves continuously through coordinate space, i.e. transitions between non-neighbouring bins do not occur for sufficiently small *τ*. Note that the number of parameters scales linearly with the number of bins in the simulation.

The trajectories we aim to analyse are initiated close to the top of the free energy barrier and, as they evolve in time, will leave the region of interest and end up in a (meta)stable basin. As a consequence, it is natural to implement absorbing boundary conditions by terminating trajectories once they leave a certain range of *q*-values. However, doing so adds new elements to the rate matrix with indices 0 and (n+1) that violate the detailed balance condition mentioned above, because once a trajectory is absorbed at the boundary it cannot return. So while transitioning to these boundary bins occurs with finite probability, $R_{i0}=R_{i,(n+1)}=0$ holds for the respective reverse transitions. The rate matrix becomes necessarily singular, and efficient treatment of the matrix exponential becomes slightly more involved. For details on this point, we refer the reader to Appendix 1.

To maximise our likelihood function *L*, we conducted an MCMC simulation that randomly displaces a single independent parameter (either $R_{ij}$ or $\ln p_{i}$) by a small amount at every step. We impose the necessary conditions expressed in Equation (8) before computing the likelihood function via Equation (7). The generation probability of such a move is symmetric and the Metropolis rule was used for the acceptance probability

(9) $$p(R_{ij}\to R_{ij}^{\prime })=\min[1,e^{\alpha (\ln L^{\prime }-\ln L)}].$$

Here, the parameter *α* corresponds to an artificial reciprocal temperature that is initially set to a low value to accelerate equilibration, allowing one to transverse a rough likelihood landscape. As the simulation progresses, we increase *α*. The sampling is then expected to relax towards a point of high likelihood, yielding good approximations for the best set of parameters consistent with all desired conditions.

## 3. Discretisation effects in artificial nucleation processes

Before applying the Bayesian analysis method to results of molecular simulations, we test it using dynamical trajectories obtained for a simple one-dimensional nucleation model. For this model, we will investigate in detail how the results of the calculation depend on parameters such as the number and width of the bins as well as the lag time. The main goal here is to find a good compromise between accuracy and computational effort.

### 3.1. Test model

Our test model consists of a one-dimensional variable, *v*, representing the volume of a vapour bubble in a liquid, evolving stochastically according to a Langevin equation on a free energy surface G(v) with diffusivity D(v). Here, multiplicative noise arises from a state-dependent D(v), so that one needs to include an appropriate drift term to preserve the equilibrium distribution proportional to $e^{-\beta G(v)}$. Specifically, we use the Itô form of the Langevin equation, and accordingly trajectories are generated with a simple Itô integrator [7]

(10) $$\begin{array}{rl} v(t+\Delta t) & =v(t)+[D^{\prime }(v)-\beta G^{\prime }(v)D(v)]\Delta t\\ & \;+g_{t}\sqrt{2D(v)\Delta t}, \end{array}$$

where Δt is the time step and both G(v) and D(v) are assumed to be continuously differentiable. Besides the thermodynamic force, $-\beta DG^{\prime }$, there is a drift term, $D^{\prime }$, originating from the state-dependent diffusivity. In the above equation, $g_{t}$ represents a random variable drawn from a Gaussian distribution with unit variance and zero mean.

The specific forms of G(v) and D(v) for our test model were chosen to mimic the behaviour of cavitation bubbles in metastable water [8]:

(11) $$\begin{array}{rl} G(v) & =4\pi \gamma_{0}r^{2}(v)+pv, \end{array}$$

(12) $$\begin{array}{rl} D(v) & =\frac{3k_{B}T}{4\eta }v. \end{array}$$

Here, $\gamma_{0}$ denotes the surface tension of the vapour–liquid interface, *p* is the pressure, $k_{B}$ is the Boltzmann constant, *T* is the temperature, and *η* is the viscosity of the liquid. The quantity $r(v)=(3v/4\pi {)}^{1/3}$ corresponds to the radius of a spherical bubble with volume *v*. For negative pressures *p*, the free energy G(v) exhibits a maximum at position

(13) $$v_{\max}=\frac{32\pi \gamma_{0}^{3}}{3|p{|}^{3}}.$$

If not explicitly stated otherwise, the following parameters were used to obtain the subsequent simulation results: surface tension $\gamma_{0}=17.09\;k_{B}T/{\mathrm{nm}}^{2}$, dynamic viscosity of the liquid medium η=1.00mPas, temperature T=296.4K and pressure p=−135MPa. Energies are given in units of the thermal energy, $k_{B}T$. The value of the surface tension $\gamma_{0}$ is based on a computational estimate for TIP4P/2005 water [22]. Note that the free energy expressed above does not include curvature effects on the surface tension [8].

We consider trajectories in a *v*-interval centred around the barrier top and selected so that the corresponding free energy, G(v), spans a few $k_{B}T$. To obtain estimates for the transition probabilities, a large number of short trajectories are initiated near the barrier top. The trajectories are advanced until they encounter one of the absorbing walls placed at the interval's boundaries. The system is propagated with a time step of Δt=1.0fs and transitions are evaluated in intervals of a selected lag time, *τ*. Coordinate bins have a constant width Δv, but the position of their centre shifts according to the current value of *v* at the beginning of a transition move. A shift of up to Δv/2 in either direction is sufficient to ensure any step starts with the trajectory at the centre of a bin in the uniformly shifted set, thereby reducing discretisation errors.

### 3.2. Discretisation parameters

**Figure 2.**
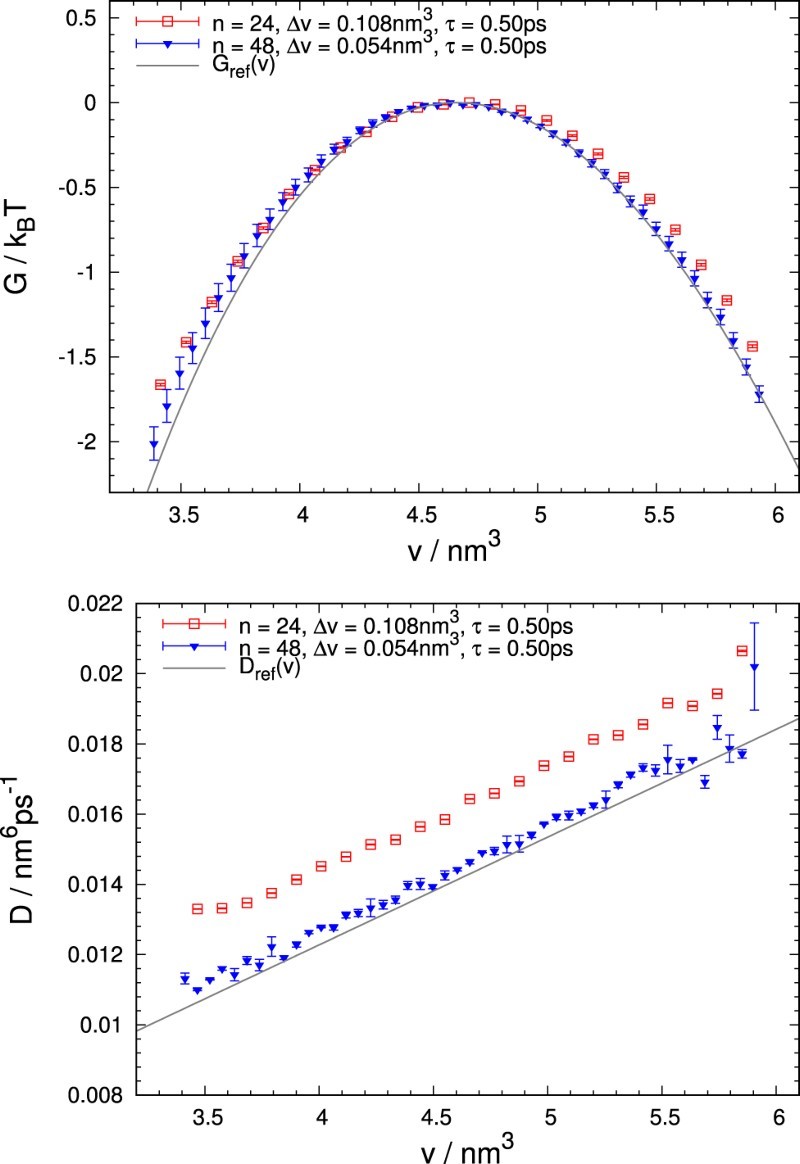
Free energy, G(v), and diffusivity, D(v), determined from $2\times 10^{4}$ trajectories generated for the test model. The results shown correspond to analyses with *n*=24 and *n*=48 bins, obtained for a lag time of τ=0.50ps. The absorbing boundary conditions were placed at the end points of an interval of width $n\Delta v=2.60\;{\mathrm{nm}}^{3}$ centred at the top of the barrier. $G_{\mathrm{ref}}$ and $D_{\mathrm{ref}}$, indicated by grey lines, are the reference functions from Equations (11) and (12), respectively. Larger errors of some data points for *n*=48 indicate an insufficient number of transitions in the respective bin.

Results obtained by analysing $2\times 10^{4}$ trajectories generated for our test model are shown in Figure 2. Henceforth, all error bars depicted correspond to standard deviations of results obtained from five independent MCMC simulations. The retrieved free energy, shown in the top panel, agrees rather well with the reference free energy G(v) even with a moderate number of discretisation bins (*n*=24). Especially around the barrier top the curvature of the free energy is reproduced well. Yet, these estimates are consistently higher than the reference line, which hints at systematic errors arising in the analysis. Results for *n*=48 bins lie significantly closer to the expected values, indicating that these deviations from the reference curve need to be attributed to discretisation errors. Similar observations apply to the estimates of diffusion coefficients, D(v), shown in the lower panel. Considerable deviations in close proximity to the boundaries appear due to the thermodynamic force term, $-\beta DG^{\prime }$: Many trajectories reach an absorbing wall before a transition event in a bin close to the barrier edge can be registered, effectively lowering the quality of near-boundary histograms and introducing larger statistical deviations. Just like the free energy, the estimated diffusion coefficients suffer from systematic discretisation errors (for *n*=24 the estimate is considerably larger than the reference curve), which decay quickly as the number of bins is increased.

The degree of agreement of the simulation results with the prescribed landscapes depends sensibly on the interplay of the discretisation parameters Δv and *τ*. As shown, for a fixed *τ* and fixed range of the reaction coordinate *v*, the quality of the estimates improves with a growing number of bins *n*. Nevertheless, increasing *n* is costly as the computational effort scales cubically with *n* due to the evaluation of a matrix exponential at every step. To obtain good numerical estimates at acceptable computational expense, it is important to make appropriate choices for the reaction coordinate spacing Δv along with the lag time *τ*. A suitable set of discretisation parameters allows one to compensate greatly for the deviations caused by a small number of bins.

**Figure 3.**
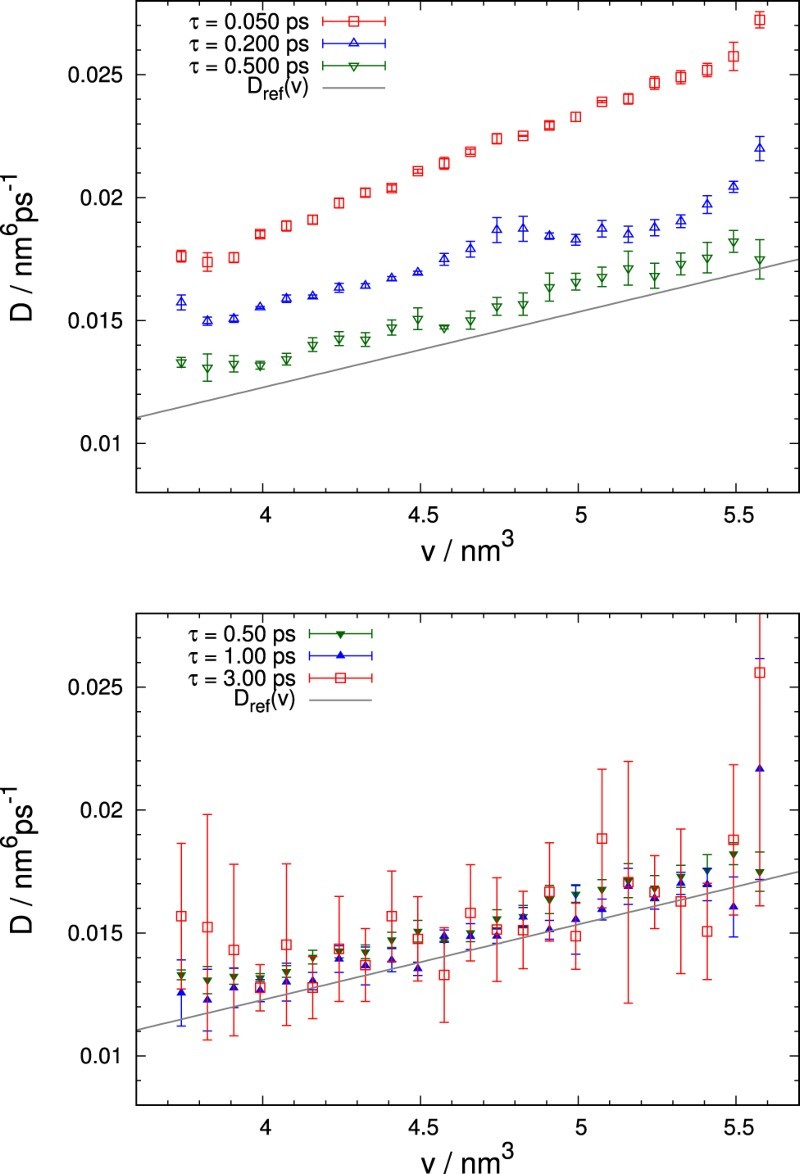
Comparison of estimates of D(v) for different lag times, *τ*, averaged over five simulations of 2000 trajectories each. The width of the *v*-interval was $n\Delta v=2.00\;{\mathrm{nm}}^{3}$ and the number of bins *n*=24. Symbols refer to the results of the calculation while the solid line indicates the reference diffusion coefficient of Equation (12). For short lag times, *τ*, results show small statistical but large systematic deviations. For large *τ* this situation is reversed.

The optimum choice of *τ* depends on the selected bin size Δv. Figure 3 demonstrates the typical behaviour of the estimated diffusion coefficient, D(v), obtained for different lag times with constant Δv and a small number of trajectories. Evidently, the estimates are strongly affected by the particular choice of *τ*: Simulations with the shortest lag time of 50fs result in severely inaccurate approximations for the diffusion coefficients, with these deviations becoming smaller for larger lag times. Since the statistical deviations indicated by the error bars are small, the errors must be due to discretisation or short-time memory effects, which become less severe for growing *τ*.

Nevertheless, *τ* cannot be made arbitrarily large either: Besides trajectories reaching the absorbing edge of the sampling range within finite time, increasing *τ* noticeably thins out the number of uncorrelated transition events from limited data. Such a decrease in informational content negatively affects the transition histograms for each bin, but especially in regions of large thermodynamic force, that is, high average velocity. This leads to large statistical errors, which are particularly apparent for τ=3ps as shown in Figure 3. It needs to be stressed, however, that even though the statistical errors are large, the results lie appreciably closer to the reference values than for short *τ*. Differences between the simulations with τ=0.50ps and 1.00ps appear to be minor, but most of the τ=1.00ps results assume values closer to the reference curve except for the rightmost points. This illustrates the statistical deterioration due to a larger thermodynamic force, inducing a tendency to skip bins, especially if they are situated close to the boundary. Otherwise, transition histograms are still sufficiently accurate to yield results with small deviations.

We underline at this point that in practical applications the free energy and especially the diffusivity are rarely known beforehand even as rough approximations. Therefore, it becomes necessary to check the self-consistency of the calculated estimates by conducting multiple simulations at progressively larger *τ* values. This procedure has the advantage that, with increasing *τ*, short-time correlations that are not captured by the simplified diffusive dynamics are effectively integrated out, thereby ensuring that the dynamics is Markovian, as required.

### 3.3. Mean first passage times

As discussed above, the lag time *τ* is a crucial parameter for an accurate retrieval of free energies and diffusion coefficients from dynamical trajectories. In the following, we will discuss an analysis based on mean first passage times (MFPT) [2,23] that helps to choose appropriate lag times to strike a good balance between systematic and statistical errors.

We consider the MFPT for trajectories started at the top of a free energy barrier. To determine the MFPT for these trajectories, one measures the time it takes to first reach a certain distance, *b*, from its initial position and then averages this time over the set of trajectories. The MFPT as a function of *b* then yields important information on the dynamics of the system as it crosses the barrier and gives an estimate for the local diffusion coefficient.

**Figure 4.**
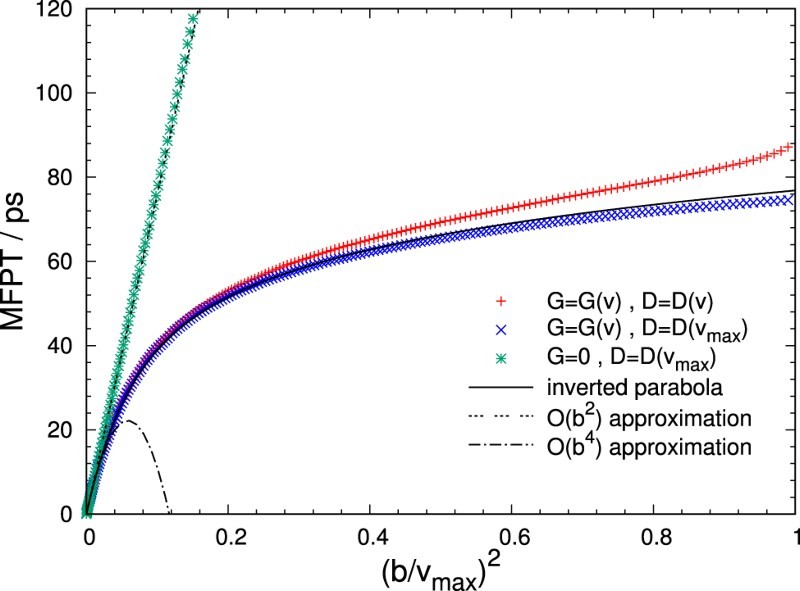
Mean first passage time MFPT as a function of $(b/v_{\max}{)}^{2}$ obtained from 5000 trajectories of our test model for a pressure of p=−135MPa. Here, $v_{\max}=4.659\;{\mathrm{nm}}^{3}$ is the position of the free energy maximum at this pressure. Results for three different cases are shown: both G(v) and D(v) obey the expressions specified in Equations (11) and (12), respectively (crosses); G(v) is variable, but the diffusion coefficient is fixed at $D(v_{\max})$ (diagonal crosses); both the free energy and the diffusion constant are fixed at $G(v_{\max})$ and $D(v_{\max})$ (stars). Furthermore, theoretical a priori estimates are shown, corresponding to diffusion on an inverted parabola approximating G(v) with constant diffusion coefficient. The quadratic order approximation of this solution corresponds to the flat free energy case.

Mean first passage times obtained from 5000 trajectories generated for our test model are shown in Figure 4 as crosses. In addition, the figure includes also results for which the diffusion coefficient (diagonal crosses) or both the diffusion coefficient and the free energy (stars) are held fixed at their values at the position of the free energy maximum, $v_{\max}$. For the case where the system evolves with constant diffusivity on a flat free energy landscape, the MFPT is simply given by $\mathrm{MFPT}(b)=b^{2}/2D$. At small distances from the barrier top the remaining two cases follow this behaviour due to an almost flat free energy, $G^{\prime }(v)\approx 0$, and small local differences in the diffusion coefficient, $D(v)\approx D(v_{\max})$. For larger values of *b*, the MFPT is primarily governed by the thermodynamic force term proportional to $G^{\prime }(v)$, the magnitude of which grows as the system moves away from the top of the barrier, decreasing the slope of MFPT(b). In comparison, including a variable D(v) only has a minor effect. The small increase of the MFPT observed in this instance with respect to the fixed diffusivity case is caused by a decrease of the diffusion coefficient as the bubble volume *v* approaches zero.

For slowly varying diffusion coefficients, it is useful to approximate the shape of the barrier as an inverted parabola around $v=v_{\max}$ with curvature $\omega =\sqrt{|d^{2}G/dv^{2}|}$. This allows one to arrive at an explicit expression for the MFPT

(14) $$\begin{array}{rl} \mathrm{MFPT}(b) & =\frac{b^{2}}{2D}{}_{2}F_{2}\left(1,1;\;2,\frac{3}{2};\;-\frac{\beta \omega^{2}b^{2}}{2}\right)\\ & =\frac{b^{2}}{2D}\left(1-\frac{\beta \omega^{2}b^{2}}{6}\right)+O(b^{6}), \end{array}$$

that closely approximates the constant diffusion case depicted in Figure 4 over the whole range shown. Here, ${}_{2}F_{2}$ denotes a generalised hypergeometric function. For a more detailed derivation of this formula, we refer to Appendix 2.

The typical bin widths Δv employed in the algorithm tend to fall in the regime where the MFPT depends quadratically on *b*. Thus, a value around $\tau =\mathrm{MFPT}(\Delta v)=\Delta v^{2}/2D(v_{\max})$ may serve as a good starting point for the discretisation, as this is the typical time scale of permanence in one bin near the free energy maximum. However, these values should be considered a lower limit of practically relevant transition lag times: The MFPT represents a measure of when the trajectory is expected to cross to an adjacent bin, but it is desirable to select a temporal discretisation that allows for even farther transitions with decent probability, in order to spread transition histograms.

## 4. Diffusivity of cavitation bubbles in water at negative pressures

We will now turn to the analysis of cavitation trajectories obtained previously using MD simulations [8]. First, we will provide a brief overview of these simulations, referring the reader to Ref. [8] for the full simulation details. Then, we will extract diffusion coefficients and free energies from these trajectories.

### 4.1. Model and simulations

In Ref. [8], the molecular mechanism for cavitation in water at negative pressures was studied using the TIP4P/2005 model of water [22]. Simulations were carried for 2000 water molecules in the isothermal-isobaric ensemble at temperature T=296.4K and pressures in the range of p=−(165⋯105)MPa in steps of 15MPa. Constant temperature and pressure were imposed with a Nosé-Hoover thermostat chain [24] in conjunction with an Andersen barostat [25]. The trajectories we analyse here were originally obtained to compute cavitation rates using a variant of the reactive flux approach [8,26].

For this system, the Gibbs free energy G(v) as a function of the largest bubble volume *v* in the system follows very closely the expression

(15) $$G(v)=4\pi r^{2}(v)\frac{\gamma_{0}}{1+2\delta /r(v)}+pv.$$

Here, we use $\gamma_{0}=20.24\;k_{B}T/{\mathrm{nm}}^{2}$ and δ=0.195nm, as obtained by fitting free energy profiles [8] harvested with a combination of umbrella sampling and a hybrid Monte Carlo scheme [27]. The above expression holds for all pressures in the range p=−(165⋯105)MPa. The surface free energy contribution, i.e. the first term on the right-hand side of Equation (15), includes a Tolman-like correction that takes the curvature dependence of the surface tension into account (compare with Equation (11)). The free energy maximum is located at

(16) $$v_{\max}=\frac{4\pi }{3}{\left(\frac{\gamma_{0}}{|p|}\right)}^{3}{\left(1-\frac{4\delta }{r_{0}}+\sqrt{1+\frac{4\delta }{r_{0}}}\right)}^{3},$$

where $r_{0}=2\gamma_{0}/|p|$ corresponds to the radius of the bubble at the free energy maximum predicted by CNT without curvature correction as in Equation (13).

### 4.2. Mean first passage times

**Figure 5.**
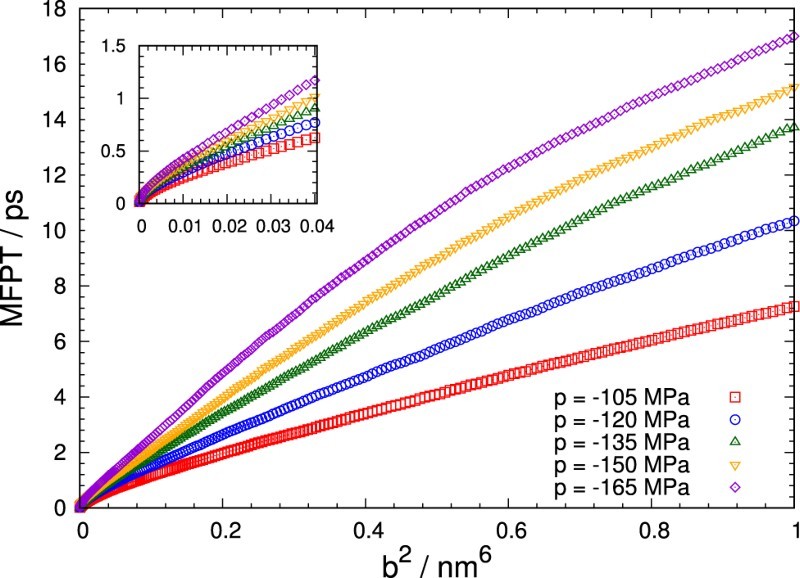
Mean first passage times at different pressures *p* averaged over 5000 MD trajectories as a function of the quadratic barrier distance $b^{2}$. Trajectories were initialised atop the barrier at $v_{\max}$.

To obtain useful estimates for the lag time, we computed the MFPT for reaching a certain distance *b* from the free energy maximum. MFPTs as a function of $b^{2}$ are shown in Figure 5 for different pressures. Since bin widths Δv are typically small, only accordingly low *b* values are relevant in practice. The MFPTs in this range are highlighted separately in the figure's inset. For all further analysis, we select bin widths for a lag time of either τ=0.40ps or τ=0.50ps based on these MFPTs.

### 4.3. Free energy and diffusivity landscapes

In the following, we will discuss the reconstruction of the free energy and diffusivity landscapes from MD data. In order to shorten the time needed to converge the MCMC procedure, the calculation was started with the analytic expression for G(v) from Equation (15) as initial values. If no such estimate were available, one could also start with the simple CNT estimate of Equation (11) or even a flat free energy profile. Diffusion coefficients D(v) were initialised as constants. During the calculation, both G(v) and D(v) evolve according to the likelihood landscape until convergence is reached. We consider a set of parameters as sufficiently converged once the likelihood function does no longer rise appreciably and only fluctuates weakly.

**Figure 6.**
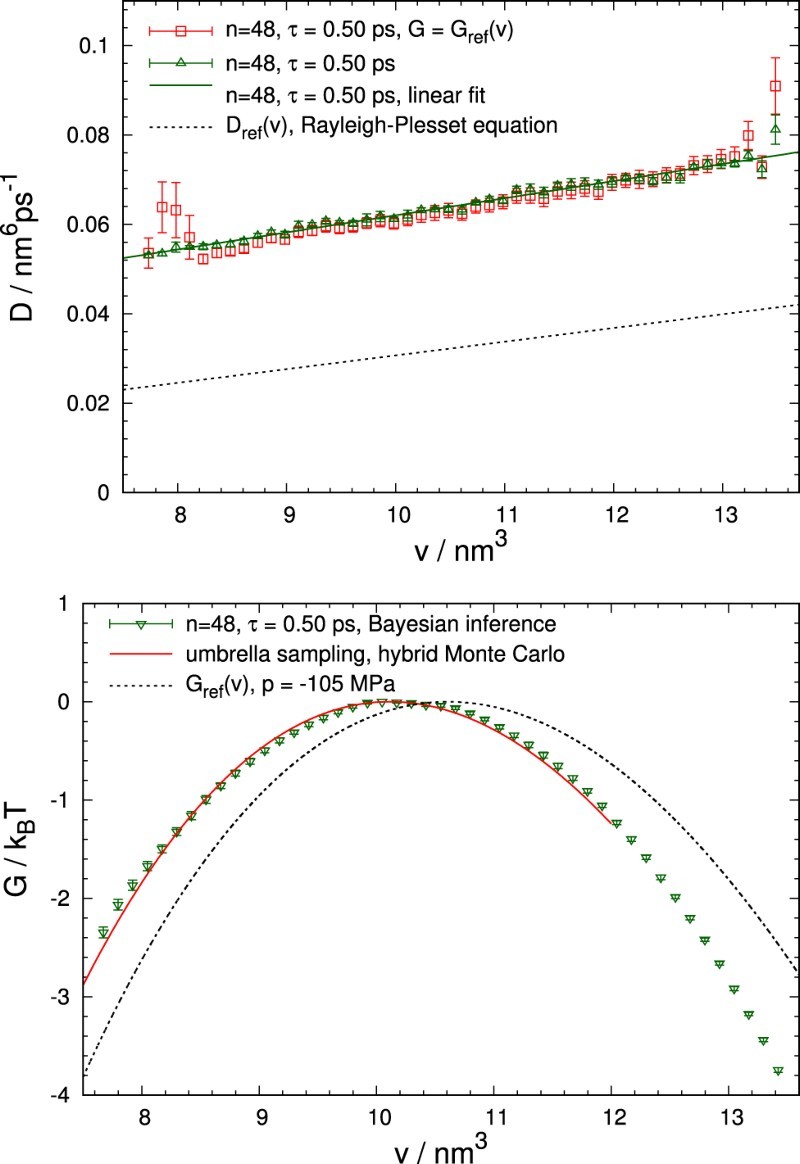
Diffusivity D(v) (top) and free energy G(v) (bottom) obtained from MD trajectories of cavitation at pressure p=−105MPa. The analysis was carried out for bin number *n*=48, sampling range $n\Delta v=6.00\;{\mathrm{nm}}^{3}$, 6500 trajectories, and lag time τ=0.50ps, amounting to approximately 1.5 times the corresponding MFPT. The diffusion coefficients are shown for both prescribed and variable free energy. In the lower panel, the reconstructed free energy G(v) (symbols), the free energy obtained directly in Ref. [8] from simulations via umbrella sampling (solid line), and the estimate of Equation (15) (dashed line) are depicted.

The diffusivity D(v), determined from MD trajectories generated at a pressure of p=−105MPa, is shown as a function of bubble volume *v* in the top panel of Figure 6 together with the prediction of the Rayleigh–Plesset equation [8], as expressed in Equation (12). Remarkably, the diffusion coefficient obtained from the Rayleigh–Plesset equation is quite close to the behaviour of the reconstructed diffusion coefficient, despite being a purely macroscopic model for the dynamics of vapour-filled bubbles based on continuum hydrodynamics.

For comparison, we also determined the diffusion coefficient with prescribed free energy profile G(v) rather than reconstructing the free energy together with the diffusivity. Results of this calculation, for which we used the free energy given by Equation (15) as a reference, are shown in the top panel of Figure 6 as square symbols. As can be inferred from the figure, the diffusion coefficients obtained with fixed and variable free energy essentially do not differ from each other and only near the boundary statistically discernible differences occur. This close agreement is particularly noteworthy when one considers that the reconstructed free energy differs notably from Equation (15), as can be seen in the bottom panel of Figure 6. Nonetheless, the computed results lie remarkably close to the respective simulation estimates obtained in Ref. [8], demonstrating the consistency of our Bayesian inference analysis.

Before examining further results pertaining to different, even lower *p* values, let us briefly comment on the particular choice of parameters. In all subsequent calculations, *n*=24 bins were used, which allow us to generate well-converged, self-consistent estimates with moderate computational effort. Our choice of τ=0.40ps for the lag time limits the range of permissible bin widths, but in return avoids excessive thinning out of the number of bin-to-bin transitions. Bin widths vary from $0.080\;{\mathrm{nm}}^{3}$ for −165MPa to $0.108\;{\mathrm{nm}}^{3}$ for −120MPa and are appropriately adapted to the corresponding MFPTs of approximately 0.30⋯0.32ps, i.e. a value a little smaller than the actually used *τ*.

**Figure 7.**
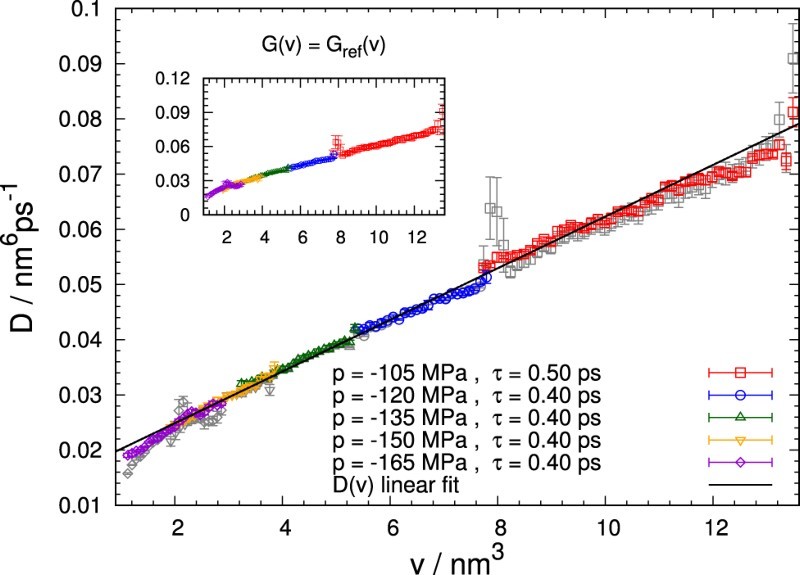
Diffusion coefficient D(v) as a function of bubble volume *v* retrieved for different pressures. Results obtained with prescribed free energy profiles are shown in the inset and, additionally, in the main plot as grey symbols. For all pressures, the obtained diffusion coefficients follow the same linear dependence on the bubble volume with slight deviations for small bubbles. A linear fit to the diffusion coefficients obtained for all pressures (solid line) yields a slope of $(4.678\pm 0.029)\times 10^{-3}\;{\mathrm{nm}}^{3}\;{\mathrm{ps}}^{-1}$ and a *D*-axis intercept of $(1.553\pm 0.099)\times 10^{-2}\;{\mathrm{nm}}^{6}\;{\mathrm{ps}}^{-1}$.

Diffusion coefficients obtained for different pressures are shown Figure 7 as a function of bubble volume. Remarkably, all diffusion coefficients lie on the same linear fit and agree well where the curves overlap. Deviations from linearity occur only for very small bubbles with a volume $v<2\;{\mathrm{nm}}^{3}$. Essentially the same results (with some exceptions at the boundaries) are obtained if the free energy is prescribed according to Equation (15) rather than optimising it together with the diffusion coefficients. Such linear behaviour of the diffusion coefficient D(v) without significant pressure dependence is predicted by the macroscopic Rayleigh–Plesset equation when augmented with appropriate thermal fluctuations. Note, however, that the diffusion coefficient derived from the Rayleigh–Plesset equation does not have a finite intercept on the *D*-axis. Its slope of $3k_{B}T/4\eta \approx 3.069\times 10^{-3}\;{\mathrm{nm}}^{3}\;{\mathrm{ps}}^{-1}$, on the other hand, evaluated for a viscosity 1.00mPas [8], differs only by about 35% from the simulation results. This observation indicates that the macroscopic Rayleigh–Plesset theory holds down to approximately nanoscopic bubbles, despite not capturing all aspects of bubble dynamics in this regime.

## 5. Conclusions

In this work, we have applied a Bayesian inference algorithm [7] to extract state-dependent diffusion coefficients and free energies from dynamical barrier crossing trajectories of nucleation processes. In particular, we have determined these quantities for cavitation occurring in liquid water at strongly negative pressures. During this process, vapour bubbles form and grow stochastically, eventually leading to the decay of the metastable liquid phase. Analysis of the time evolution of the bubble volume, which has previously been shown to be a good reaction coordinate for this process [8], yields the respective diffusivity and free energy profile on the nucleation barrier.

In applying the Bayesian inference algorithm, which is based on a discretisation of the Fokker–Planck equation, it is important to choose appropriate discretisation parameters to yield sufficient accuracy at an affordable computational cost. For the cavitation problem studied here, discretising the reaction coordinate into several dozens of bins combined with an appropriate lag time, estimated using a first passage time analysis, yields satisfying results both for the diffusion coefficient as well as for the free energy.

Our results indicate that the method should be generally applicable to nucleation processes provided the dynamics of the selected reaction coordinate is Markovian, as assumed in CNT. It should be noted, however, that the existence of a good reaction coordinate already implies at least approximately Markovian dynamics [28]. As a practical example, Bayesian inference can be used to analyse trajectories generated in the seeding approach to nucleation processes [6]: Applied to crystallisation trajectories, such a calculation would provide the attachment rate, the Zeldovich factor as well as the size of the critical cluster needed for the calculation of crystallisation rates. Furthermore, computing the diffusion coefficient as a function of nucleus or bubble size allows one to verify the often made assumption of constant diffusivity in the barrier region.

Knowledge of the diffusion coefficient as a function of the reaction coordinate is not only important for estimating nucleation rates in the framework of CNT but also provides useful information on the molecular mechanism controlling the growth and decay of nuclei in the early stages of nucleation. In the case of cavitation in water under tension, for instance, our analysis of dynamical trajectories has shown that the dependence of the diffusivity on the bubble volume is basically consistent with predictions based on the Rayleigh–Plesset equation [8]. Residual discrepancies between our estimates and theory also hint at a curvature-dependent viscosity, as originally introduced by Dzubiella [29,30]. To examine this notion, it is necessary to investigate further into the low-volume regime, where departure from the postulated linear behaviour is already apparent by the diffusivity profiles shown here. Nonetheless, our results suggest that the mechanism posited in Rayleigh–Plesset theory is essentially correct even on the nanoscale, implying that the viscosity of the liquid is the main factor to determine the dynamics of bubble growth and decay in water under strong tension.

## Appendix 1. Appendix 1. Transition probabilities in presence of absorbing boundaries

In this appendix, we will demonstrate an efficient way to calculate the matrix exponential $e^{\tau R}$ of a rate matrix *R* in a system with absorbing boundary conditions. As noted in Section 2, absorbing boundary conditions violate detailed balance, complicating the diagonalisation of *R*. Without this complication, one could follow the same approach as used by Hummer in Ref. [7]: Applying a similarity transformation to *R* one arrives at $R_{ij}^{\prime }=p_{i}^{-1/2}R_{ij}p_{j}^{1/2}$, which is a symmetric matrix if detailed balance holds. Symmetric matrices can be diagonalised efficiently and in a computationally stable fashion by orthogonal transformations, e.g. by utilising the QR algorithm [31]. Then, one calculates the exponentials of the computed eigenvalues and reverses all similarity transformations, obtaining the final result. Although one cannot avoid that such a computation scales like $O(n^{3})$, where *n* is the number of (non-absorbing) bins, this procedure is much more efficient than working out the exponential via its series expansion, for instance.

To achieve similar computation speeds for absorbing boundary conditions, we aim to adapt the described approach to this particular case of broken symmetry. Consider the slightly larger (n+2)×(n+2) matrix *R*. Indices 0 and (n+1) now pertain to absorbing boundaries, and the respective columns strictly vanish. For clarity, we show an example for a system with *n*=5 inner bins, where asterisks indicate non-vanishing elements:

$$\left(\begin{array}{ccccccc} 0 & * & 0 & 0 & 0 & 0 & 0\\ 0 & * & * & 0 & 0 & 0 & 0\\ 0 & * & * & * & 0 & 0 & 0\\ 0 & 0 & * & * & * & 0 & 0\\ 0 & 0 & 0 & * & * & * & 0\\ 0 & 0 & 0 & 0 & * & * & 0\\ 0 & 0 & 0 & 0 & 0 & * & 0 \end{array}\right).$$

Note that although this matrix does not satisfy detailed balance as a whole, the submatrix ${\bar{R}}_{ij}=R_{ij}$, where i,j∈{1,…,n} does. We can utilise the diagonalisation procedure outlined above on this submatrix, and all orthogonal transformations ${\bar{U}}_{k}$ accumulated in the course of this computation are summarised as a single one denoted by $\bar{U}=\prod_{k}{\bar{U}}_{k}$. Furthermore, we expand this transformation in a block diagonal fashion, so that it can be applied to *R* as a whole, i.e.

(A1) $$U_{ij}=\left\{\begin{array}{ll} {\bar{U}}_{ij} & \mathrm{if}\;i,j\in \{1,\ldots ,n\},\\ \delta_{i0}+\delta_{i,(n+1)} & \mathrm{else}, \end{array}\right.$$

where $\delta_{ij}$ is the Kronecker-delta. Doing so yields a matrix *A* of the form

(A2) $$A=U^{T}(\tau R)U=\left(\begin{array}{ccc} 0 & {\vec{a}}_{0} & 0\\ \vdots & D & \vdots \\ 0 & {\vec{a}}_{n+1} & 0 \end{array}\right).$$

Here, ${\vec{a}}_{0}$ and ${\vec{a}}_{n+1}$ denote row vectors, $d_{ii}$ henceforth indicates the elements of diagonal matrix *D*, corresponding to the eigenvalues of $\bar{R}$, and $U^{T}$ signifies the transpose of *U*. An expression for the *k*th power of *A* can be derived inductively and one obtains

(A3) $$A^{k}=\left(\begin{array}{ccc} 0 & a_{0i}d_{ii}^{k-1} & 0\\ \vdots & D^{k} & \vdots \\ 0 & a_{n+1,i}d_{ii}^{k-1} & 0 \end{array}\right)\;\forall \;n>0.$$

Inserting this expression into the definition of the matrix exponential as series yields

(A4) $$e^{A}=\sum_{k=0}^{\infty }\frac{A^{k}}{k!}=\left(\begin{array}{ccc} 1 & a_{0i}d_{ii}^{-1}(e^{d_{ii}}-1) & 0\\ 0 & e^{D} & 0\\ 0 & a_{n+1,i}d_{ii}^{-1}(e^{d_{ii}}-1) & 1 \end{array}\right),$$

where we used that

(A5) $$\begin{array}{rl} \sum_{k=1}^{\infty }\frac{a_{ji}d_{ii}^{k-1}}{k!} & =\frac{a_{ji}}{d_{ii}}\left[\left(1+\sum_{k=1}^{\infty }\frac{d_{ii}^{k}}{k!}\right)-1\right]\\ & =\frac{a_{ji}}{d_{ii}}(e^{d_{ii}}-1). \end{array}$$

The last step consists of undoing the orthogonal transformations, so that one finally obtains $e^{\tau R}=Ue^{A}U^{T}$. Overall, this procedure avoids the initial problems of absorbing rate matrices, and increases the computational effort only by an insignificant amount, so that it is just as efficient and stable as the original method for symmetric matrices.

## Appendix 2. Appendix 2. MFPT on parabolic free energy barriers

In the following, we derive Equation (14) as an approximation for the mean first passage time on a barrier. More specifically, we consider the MFPT of trajectories started at the top of a parabolic free energy barrier $G(q)=-(1/2)\omega^{2}q^{2}$ and evolving given a constant diffusion coefficient, *D*. As a special case of the general formula derived by Schulten et al. [32], the MFPT in the presence of a constant diffusion coefficient can be conveniently expressed as

(A6) $$\text{MFPT}(x,b)=\frac{1}{D}\int_{x}^{b}dy\;e^{\beta G(y)}\int_{a}^{y}dz\;e^{-\beta G(z)}.$$

Here, *x* denotes the initial position of a trajectory, *b* is the position of the absorbing wall, where trajectories are terminated and *a* corresponds to the location of a reflective barrier, which ensures that the MFPT remains finite. Without imposing this second boundary, a trajectory could evolve towards either side of the free energy parabola.

Starting our trajectories at the maximum of G(q), we set *x*=0 and select also *a*=0, which is equivalent to putting another absorbing wall at position −b, symmetrically located on the other side of the barrier. For the special case of a parabolic energy landscape, the inner integral can be solved easily in terms of the imaginary error function, yielding

(A7) $$\int_{0}^{y}dz\;e^{\beta \omega^{2}z^{2}/2}=\sqrt{\frac{\pi }{2\beta \omega^{2}}}\text{erfi}\left(\sqrt{\frac{\beta \omega^{2}}{2}}y\right).$$

Furthermore, we use that

(A8) $$\int dz\;e^{-z^{2}}\text{erfi}(z)=\frac{1}{\sqrt{\pi }}z^{2}{}_{2}F_{2}\left(1,1;\;2,\frac{3}{2};\;-z^{2}\right),$$

where ${}_{2}F_{2}$ is a generalised hypergeometric function. From Equations (A7) and (A8) one arrives directly at an explicit solution for the MFPT as reported in Equation (14)

(A9) $$\begin{array}{rl} \text{MFPT}(0,b) & =\frac{b^{2}}{2D}{}_{2}F_{2}\left(1,1;\;2,\frac{3}{2};\;-\frac{\beta \omega^{2}b^{2}}{2}\right)\\ & =\frac{b^{2}}{2D}\sum_{k=0}^{\infty }\frac{(-\beta \omega^{2}b^{2}{)}^{k}}{(k+1)(2k+1)!!}\\ & =\frac{b^{2}}{2D}\left(1-\frac{\beta \omega^{2}b^{2}}{6}+\cdots \right). \end{array}$$

This equation may also serve to obtain the diffusion coefficient at the top of free energy barriers from measurements of the MFPT. Diffusivity estimates of these positions evaluated through Equation (A9) are quantitatively consistent with the ones given in the main text [8].
